# A Visualization Tool to Analyse Usage of Web-Based Interventions: The Example of Positive Online Weight Reduction (POWeR)

**DOI:** 10.2196/humanfactors.4310

**Published:** 2015-05-19

**Authors:** Emily Julia Arden-Close, Emily Smith, Katherine Bradbury, Leanne Morrison, Laura Dennison, Danius Michaelides, Lucy Yardley

**Affiliations:** ^1^ Department of Psychology Faculty of Science and Technology Bournemouth University Poole United Kingdom; ^2^ Academic Unit of Psychology University of Southampton Southampton United Kingdom; ^3^ Department of Electronics and Computer Science University of Southampton Southampton United Kingdom

**Keywords:** data visualisations, usage, Web-based interventions

## Abstract

**Background:**

Attrition is a significant problem in Web-based interventions. Consequently, this research aims to identify the relation between Web usage and benefit from such interventions. A visualization tool has been developed that enables researchers to more easily examine large datasets on intervention usage that can be difficult to make sense of using traditional descriptive or statistical techniques alone.

**Objective:**

This paper demonstrates how the visualization tool was used to explore patterns in participants’ use of a Web-based weight management intervention, termed "positive online weight reduction (POWeR)." We also demonstrate how the visualization tool can be used to perform subsequent statistical analyses of the association between usage patterns, participant characteristics, and intervention outcome.

**Methods:**

The visualization tool was used to analyze data from 132 participants who had accessed at least one session of the POWeR intervention.

**Results:**

There was a drop in usage of optional sessions after participants had accessed the initial, core POWeR sessions, but many users nevertheless continued to complete goal and weight reviews. The POWeR tools relating to the food diary and steps diary were reused most often. Differences in participant characteristics and usage of other intervention components were identified between participants who did and did not choose to access optional POWeR sessions (in addition to the initial core sessions) or reuse the food and steps diaries. Reuse of the steps diary and the getting support tools was associated with greater weight loss.

**Conclusions:**

The visualization tool provided a quick and efficient method for exploring patterns of Web usage, which enabled further analyses of whether different usage patterns were associated with participant characteristics or differences in intervention outcome. Further usage of visualization techniques is recommended to (1) make sense of large datasets more quickly and efficiently; (2) determine the likely active ingredients in Web-based interventions, and thereby enhance the benefit they may provide; and (3) guide in designing (or redesigning) of future interventions to promote greater use and engagement by enabling users to easily access valued intervention content/tools.

**Trial Registration:**

International Standard Randomized Controlled Trial Number (ISRCTN): 31685626; http://www.isrctn.com/ISRCTN31685626 (Archived by WebCite at http://www.webcitation.org/6YXYIw9vc).

## Introduction

Web-based interventions for weight management (weight loss or maintenance) have grown in popularity in recent years. There is evidence that such interventions lead to meaningful weight loss [1], particularly relative to no-intervention control groups or minimal interventions [2]. However, attrition is typically high in Web-based interventions [3-5].

In any longitudinal eHealth study, there are two different types of attrition, namely, dropout attrition, or losing participants to follow-up; and nonusage attrition (not using the intervention or low usage of the intervention). Determining nonusage and dropout attrition is an essential part of analysis of Web-based interventions, as the attrition curve may indicate the underlying cause of attrition [3]. For example, there may be steady attrition, with a consistent proportion of users discontinuing usage. Alternatively, there may be an initial phase where usage is high, followed by rapid attrition, after which a stable group of regular users remains. Further, even among regular users, some Web pages are used by almost all users who log on to the website, whereas others are never used. Although higher use of website features may be associated with weight loss, it is not clear which features improve this effect or reduce attrition [5]. It is also possible that not all users may need to complete an Internet intervention to obtain positive results—different doses may be necessary for different people [6].

Several recent studies have attempted to identify the relationship between Web usage and benefit from weight management interventions. For example, Funk and colleagues [7] categorized users of a Web-based weight loss intervention as having “consistent usage,” “some usage,” or “minimal usage.” The mean weight change was significantly higher in the “consistent usage” category, and significantly more consistent users maintained clinically important weight loss than those in the other groups. Within Internet interventions, more logins, weight and exercise entries, and use of additional features of the website after weight entry have been associated with better weight outcomes [7,8]. More specifically, use of website feedback features, such as progress charts, have been shown to be the best predictors of initial 6-month weight loss, whereas social support features, such as Web chats and participant profiles, have been related to weight maintenance at 12 months [9]. Recently, greater use of a weight tracker was associated with greater weight loss [10]. However, no study has assessed in detail whether certain Web pages are more frequently used than others, or whether certain groups of people are more likely to use particular pages. This would enable researchers to refine the content of their Web-based interventions, for example, to enable easier access to the most useful Web pages, or encourage greater use of useful but underused Web pages by identifying and addressing barriers to usage.

Positive Online Weight Reduction [11] was developed as a Web-based weight management intervention for use in primary care that aimed to result in sustainable weight loss. It was tested in a feasibility trial that consisted of 4 groups, namely: Web only, Web plus basic nurse support, Web plus regular nurse support, and usual care, to assess the extent to which weight loss was maintained at 12-month follow-up. It was designed to provide support for self-management of weight based on either a low-calorie or low-carbohydrate eating plan. Analysis revealed that average website usage, defined as duration of page viewing, was similar across the intervention arms, but extremely variable within groups. Although participants completed a mean of nine goal and weight reviews, this ranged from none to 43 completed during the 12-month trial.

Usage log data have been used to examine the relationship(s) between use of specific intervention components and subsequent outcomes/effectiveness [12-14]. Such analyses can reveal useful insights about the impact and relevance of particular components over the time course of an intervention. However, such analyses typically rely on making a priori assumptions about the specific intervention components that are expected to have an effect on uptake, adherence, or outcomes. By contrast, visualizations use aspects of exploratory sequencing techniques to summarize and plot the participant’s usage of every intervention component over time [15].Using visual analysis allows differences in usage to emerge from the data and ensures that unanticipated relationships between usage and outcomes are not overlooked. Freely available visualization tools have been developed and argued to be useful for detecting patterns of usage and how they vary across individuals/groups; detecting usability or content issues, and thereby enable researchers to edit content for use in future Web-based interventions; and performing exploratory analysis to support the design of statistical queries to summarize data regarding whether use of particular pages is related to benefit [15].

Existing visualization tools provide a useful means to explore each individual participant’s usage of an intervention, or particular aspects of all participants’ usage of an intervention (such as days/dates of logins, start and end points of each login). However, to our knowledge, these tools do not allow for a detailed comparison of how all components of an intervention have been used by all participants within one sequence plot. Our research team has therefore developed a visualization tool to examine each individual participant’s temporal usage of a Web-based intervention by illustrating what pages they have viewed, for how long, and in what order. Usage sequences for each individual are stacked within one visualization plot to facilitate comparison across all participants. This makes analysis quicker and easier compared with standard data analysis.

This paper first describes how the visualization tool works. We then illustrate the insights the visualization tool can provide by a detailed analysis of usage of the Positive Online Weight Reduction (POWeR) intervention. This analysis had three main aims, which were realized using the visualization tool.

- Examine patterns of Web usage to identify the following:
  - At what point usage of POWeR drops off;
  - Whether participants accessed both the core and optional contents of the intervention; and
  - What information, advice, and tools were reused after their initial presentation;
- Carry out a moderator analysis of patient characteristics related to Web usage; and
- Determine whether usage of specific intervention pages and sections were related to weight change.

## Methods

### Design

As reported elsewhere [11], a randomized nonblinded feasibility trial of a Web-based weight management intervention (POWeR) for obese patients in primary care was used to compare 4 parallel groups: usual care, website only, website with basic nurse support, and website with regular nurse support. The trial was approved by the UK National Health Service National Research Ethics Service, and was registered with Current Controlled Trials (ISRCTN 31685626).

### Participants and Procedures

Participants were recruited between May 2011 and December 2012 from five general practices in southern England. Inclusion criteria included being aged over 18, and having a body mass index (BMI)of 30 or more (or 28 with hypertension, hypercholesterolemia, or diabetes) documented in medical records. Exclusion criteria included being pregnant or breastfeeding, having current major mental or physical health problems, or self-reported inability to walk 100 m. Participants were followed up at 6 months and 1 year.

### Intervention

The POWeR intervention [11] consisted of 12 weekly online sessions, in which users were taught active cognitive and behavioral self-regulation techniques (eg, POWeR Tools) and provided with evidence for their effectiveness and examples of how other users had successfully used them. The sessions did not differ between groups. Session 1 provided an overview of the intervention, advice on choosing the low-calorie or low-carbohydrate eating plan, helped users to set eating goals and plan how to implement them, asked users to identify personal reasons for losing weight, and explained how to use weekly weighing as a form of self-monitoring. All subsequent sessions began by asking the users to enter their current weight and report how often they had achieved each of the goals set the previous week (goal and weight review). Following this, users received automated advice based on their progress, and were able to set new goals and plans. This advice did not differ between groups. Session 2 covered getting support from the website (eg, setting automated motivational messages), friends and family, and the nurse. Session 3 helped users choose and implement a physical activity plan (walking or mixed physical activity). Sessions 1-3 were defined as core sessions, and became available weekly in sequence. After completing the first three sessions, users could then choose any one optional session each week after their goal and weight reviews from the following selection: cravings, slipups, stretching physical activity, tough times (emotional eating), busy lives (eating when busy), setting up your environment (environment restructuring), alcoholic and nonalcoholic drinks, eating out, and maintaining weight loss. The final session was a review. In addition to the new weekly sessions, users could also reaccess content from previous sessions at any time via the main home page, using either their POWeR Tools or a weight graph plotting their progress.

### Data Collection and Analysis

**Figure 1:**
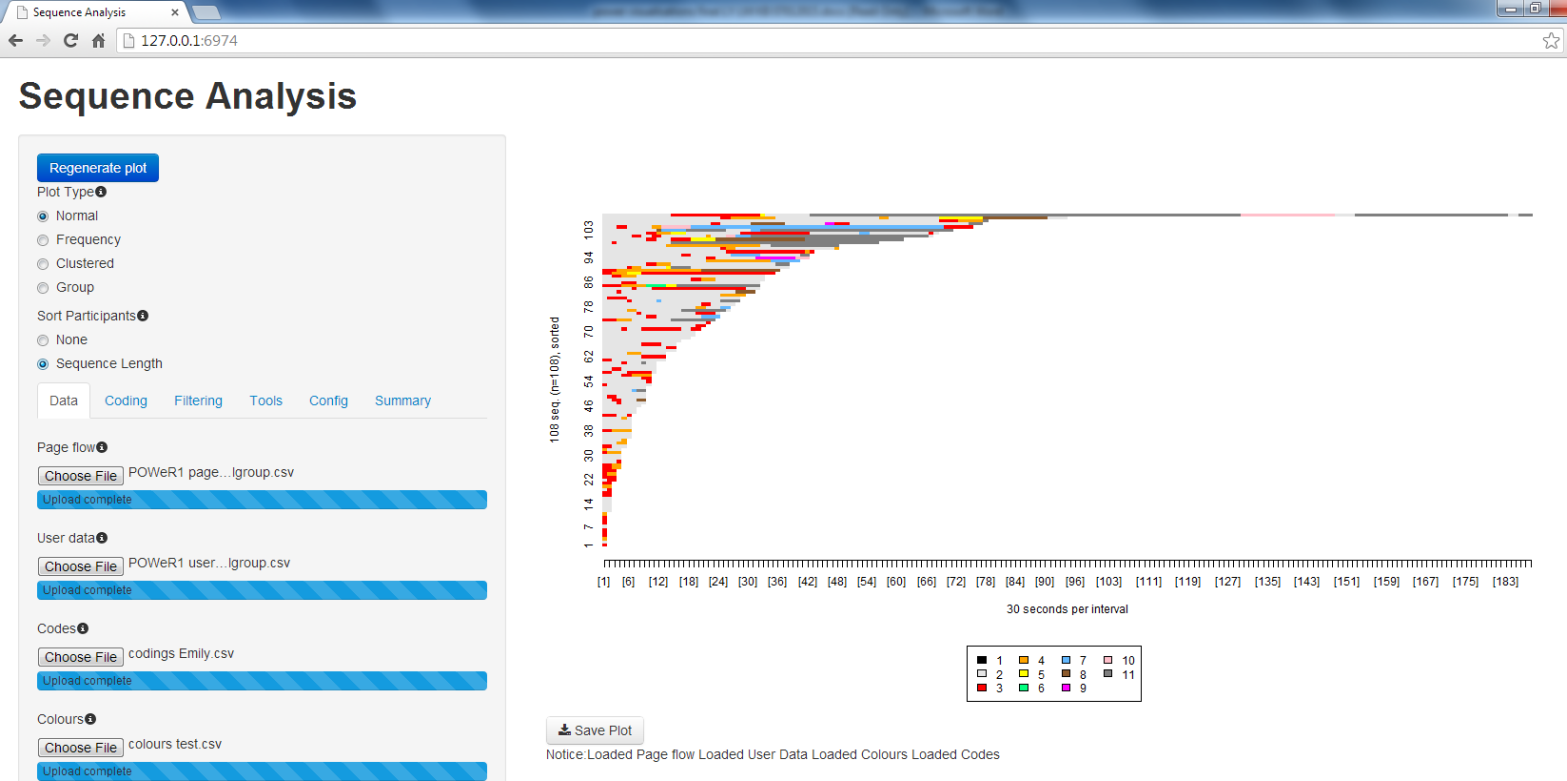
Screenshot of the visualization tool.

All data were stored using the LifeGuide intervention authoring software [16], an online software that enables researchers to create Web-based interventions. This software automatically captures data regarding all Web pages accessed, and length of time spent viewing each Web page. A visualization tool was created using R to enable us to determine patterns of Web usage. The tool enables researchers to visually compare when particular parts of the intervention were viewed, for how long, and in what order, across all participants. A Web-based interface for the visualization tool was developed using the Shiny Web application for R (Figure 1). A user guide for the visualization tool will be made available shortly, and both the tool and user manual will be made available free of charge via the LifeGuide website.

In brief, to run the tool, one needs to feed it 4 types of files: a page flow file (which shows the order in which participants have looked at pages and the time they have spent on them); a user data file (which contains data on participant characteristics and outcomes or data participants have entered into the intervention), a coding file (which assigns each intervention page a numerical code), and a color file (which assigns each intervention page code a specific color). At the top of all the interfaces, there is the option to sort participants by sequence length (the amount of time a participant has spent viewing the intervention) and choose the type of visualization plot (Table 1) the viewer wants to see.

**Table 1:** Different types of plots shown in a visualization.

Plot type	What it shows
Normal	Default option, shows which pages were viewed by each individual participant, in which order
Frequency	Shows usage by all participants by groups of pages, so the researcher can see which groups of pages are most used
Clustered	Groups participants into statistically similar usage patterns
Group	Allows you to see two or more visualizations next to each other, split into different types of usage patterns or users

The visualization can be filtered based on variables in the dataset (eg, user characteristics or outcomes) or which groups of pages users have/have not seen. If you have run a visualization that you want to follow-up on through statistical analysis, the tool can create an Excel file (Microsoft, Redmond, WA, USA) that lists details of all users who have seen a particular group of intervention pages.

### Statistical Data Analysis

Data analysis for the moderators (use of the optional sessions, food diary, and steps diary) was carried out using SPSS (SPSS Inc, Chicago, IL, USA).

## Results

### Patterns of Web Usage

Overall, 195 participants consented to take part in the feasibility trial of POWeR; 16 were enrolled at a general practice, but never used the website, and therefore, were not randomized. Participants assigned to usual care (n=43) did not have access to the website after completing questionnaires, and their data were therefore not used. There were 4 participants who went online and were assigned to a group, but never used a session. To analyze Web usage, data from the 132 participants who had viewed at least one page of a session, which comprised the groups “Web only,” “Web + basic nurse support,” and “Web + regular nurse support” were included.

Participant characteristics for the overall sample are presented in Table 2. they are not broken down by group as this information is reported in the main power paper [11].

To analyze patterns of POWeR usage, we first carried out broad-level visualizations of how participants used the entire intervention and main components of interest (eg, core versus optional sessions), followed by more fine-grained visualizations of regularly used components (eg, eating plan tools) and subsequent statistical analyses.

**Table 2:** Baseline characteristics of participants.

Participant characteristic	Mean (SD)
Age (years)	51.56 (12.96)
Age left education (years)	17.82 (2.93)
Body mass index (kg/m^2^)	35.49 (5.70)
Weight (kg)	100.66 (21.02)
Male, n (%)	46 (33.8)

### Usage of the Core and Optional Sessions

**Figure 2:**
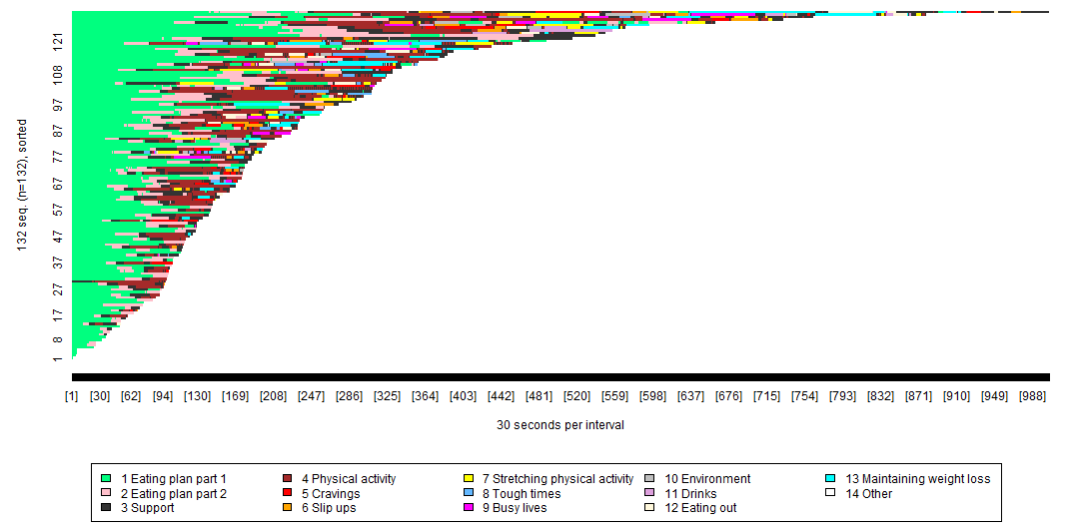
Visualization of POWeR usage of sessions by all intervention participants.

Usage of the core and optional sessions is presented in Figure 2, with each color representing a separate group of pages. For example, the light green shows usage of the first part of the eating plan pages (which introduced the eating plans), and the dark gray shows usage of the support pages. The x-axis shows the length of time spent viewing each group of pages, broken down into blocks of 30 seconds. The y-axis can be thought of as a number of lines, each representing a specific participant. Participants are presented in order of how long they spent on the intervention, with those who spent less time nearer the bottom, and those who spent more time nearer the top.

It can be seen from Figure 2 that the core eating plan session (part 1, light green; and part 2, pink) was the most widely used, followed by the core sessions on “support” (session 2, dark gray) and “physical activity” (session 3, brown). Table 3 provides a precise breakdown of the proportion of participants accessing each POWeR session (core and optional). Two thirds of the participants accessed all the core sessions. However, each optional session (except the final review session, which was made compulsory) was accessed by less than 1 in 4 of the participants. A total of 30 participants (30/132, 23%) used all the core sessions but no optional sessions. Later sessions (eg, 7-11) were viewed by only 48 participants (48/132, 36%). This contrasted with an average of 8.62 (SD 10.46) goal and weight reviews per participant (range, 0-43).

**Table 3:** Numbers (and percentages) of participants who used the core and optional sessions.

Session number	Session description	Participants who viewed at least one page of the session n (%)
1	Eating plan part 1^a^	132 (100)
1	Eating plan part 2^a,b^	120 (90.9)
2	Support^a,c^	104 (78.7)
3	Physical activity^a,c^	90 (68.1)
4	Cravings	28 (21.2)
5	Slipups	32 (24.2)
6	Stretching physical activity	25 (18.9)
7	Tough times	21 (15.9)
8	Busy lives	19 (14.3)
9	Setting up your environment	13 (9.8)
10	Drinks	13 (9.8)
11	Eating out	24 (18.1)
12	Maintaining weight loss^d^	36 (27.2)

^a^Core sessions

^b^Eating plan part 1 and part 2 are both part of session 1. They are presented separately to show the points during session 1 at which participants dropped out.

^c^The sessions are presented in the order in which they were listed.

^d^This session was made compulsory.

To further explore patterns of drop out, we used the visualization tool to compare the proportion of participants dropping out at different points during the first session. This revealed that 100% of participants (132/132) used part 1 of session 1, 120/132 (91%) used part 2 of session 1, and 115/132 (87%) completed session 1 (reached the last page). Separate visualizations were also produced for each trial arm (Web only, Web + basic nurse support, and Web + regular nurse support), but revealed no meaningful and substantial differences in attrition between groups.

**Figure 3:**
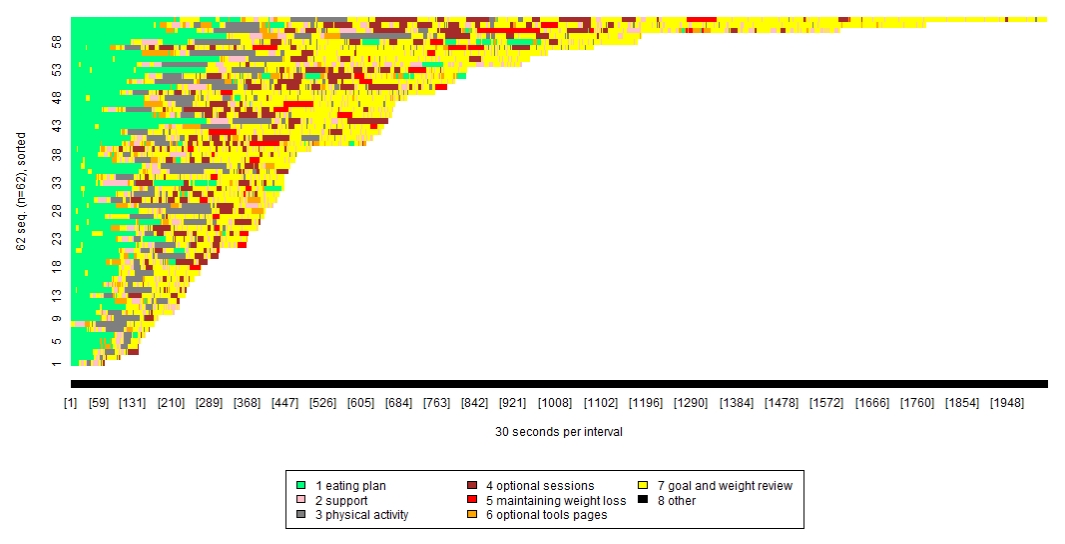
Visualization of POWeR usage by participants who used the optional sessions.

To further explore how the optional POWeR sessions were used, we filtered the visualization plots to only contain participants who accessed at least one of the optional sessions (Figure 3). This showed that after completion of the initial core sessions, 62/132 participants (47%) accessed both the goal and weight reviews (yellow) and the optional sessions (brown) whereas 58/132 participants (44%) continued to access the goal and weight reviews but not the optional sessions. Four (of 132) participants (3%) did not use either the goal and weight reviews or the optional sessions following completion of the core sessions.

In Figure 3, each color represents a separate group of pages. For example, the green shows usage of the eating plan pages, and the yellow shows usage of the goal and weight reviews pages. The x-axis shows the length of time spent viewing each group of pages, broken down into blocks of 30 seconds. The y-axis can be thought of as a number of lines, each representing a specific participant. Participants are presented in order of how long they spent on the intervention, with those who spent less time nearer the bottom, and those who spent more time nearer the top.

Figure 3 shows that 58/132 (44%) participants used the optional sessions. It also shows that the most frequently viewed pages were those relating to part 1 of the eating plan session and the goal and weight review, whereas the optional sessions and optional tools pages were not widely used.

### Repeated Use of POWeR Tools

**Figure 4:**
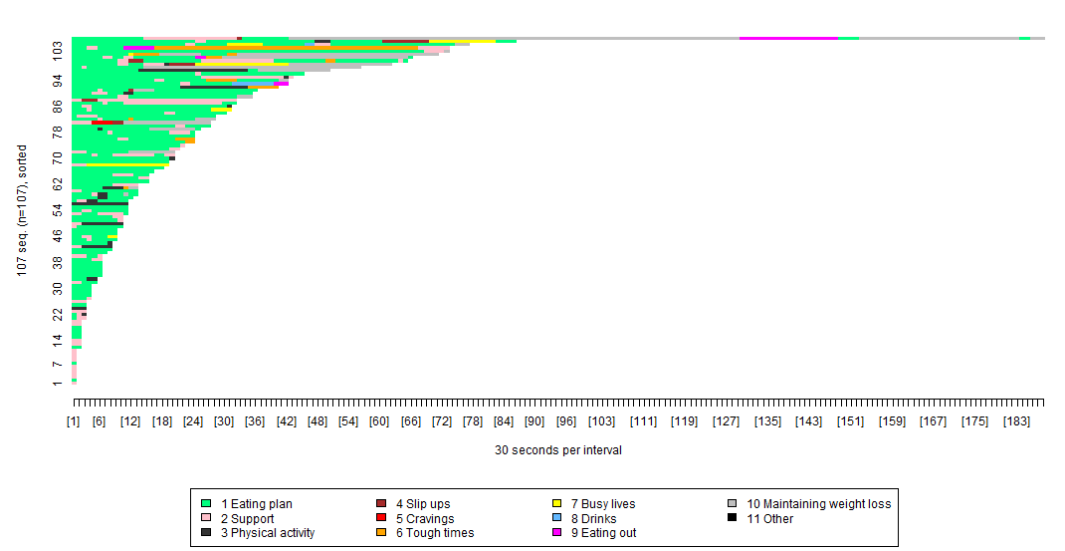
Visualization of participants’ repeated use of optional tools pages.

There were 107 participants who reused at least one of the POWeR tools, as shown in Figure 4. These data are broken down as shown in Table 4.

In Figure 4, each color represents a separate group of pages. For example, the green shows usage of the eating plan pages, and the pink shows usage of the support pages. The x-axis shows the length of time spent viewing each group of pages, broken down into blocks of 30 seconds. The y-axis can be thought of as a number of lines, each representing a specific participant. Participants are presented in order of how long they spent on the intervention, with those who spent less time nearer the bottom, and those who spent more time nearer the top.

As shown in Figure 4, the POWeR tools participants reused most pages related to the eating plan (green), support (pink), and physical activity plan (dark gray). Very few participants reused the POWeR tools pages that are related to the optional sessions.

**Table 4:** Numbers of participants who reused POWeR tools.

Tool topic	Numbers viewed n (%)
Eating plan	91 (68.9)
Support	68 (51.5)
Physical activity plan	21 (15.9)
Slipups	7 (5.3)
Cravings	1 (0.8)
Tough times	10 (7.5)
Busy lives	7 (5.3)
Drinks	2 (1.5)
Eating out	4 (3.0)
Maintaining weight loss	17 (12.8)

**Figure 5:**
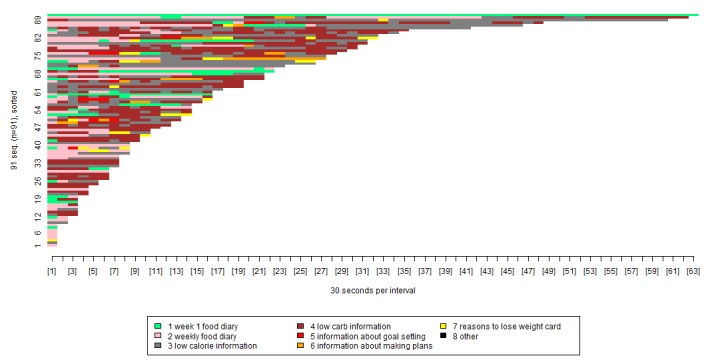
Visualization of participants’ repeated use of eating plan tools.

We used the visualization tool to provide a detailed breakdown of the most regularly reused eating plan tools (Figure 5).

In Figure 5, each color represents a separate group of pages. For example, the pink shows usage of the weekly food diary, and the yellow shows usage of the reasons to lose weight card. The x-axis shows the length of time spent viewing each group of pages, broken down into blocks of 30 seconds. The y-axis can be thought of as a number of lines, each representing a specific participant. Participants are presented in order of how long they spent on the intervention, with those who spent less time nearer the bottom, and those who spent more time nearer the top.

As shown in Figure 5, the specific tools that appeared to be reaccessed most often were those relating to the weekly food diary (light pink), and information about eating plans (eg, lists of foods that were low/high in calories or carbohydrates—gray and dark red).

The patterns observed in Figure 5 were used to provide a more precise breakdown of the proportions of participants viewing each of the eating plan tools. This confirmed that over 40% of the participants viewed the weekly food diary (76/132, 57.5%) and information about the low-calorie (71/132, 53%) and low-carbohydrate eating plans (57/132, 43%; Table 5).

**Table 5:** Numbers (and percentages) of participants who reused the eating plan tools.

Eating plan topic	Code	Numbers viewed n (%)
Week 1 food diary	1	29 (21.9)
A weekly food diary	2	76 (57.5)
Low-calorie information	3	71 (53.7)
Low-carbohydrate information	4	57 (43.1)
Information about goal setting	5	9 (6.8)
Information about making plans	6	14 (10.6)
My reasons to lose weight card	7	18 (13.6)

**Figure 6:**
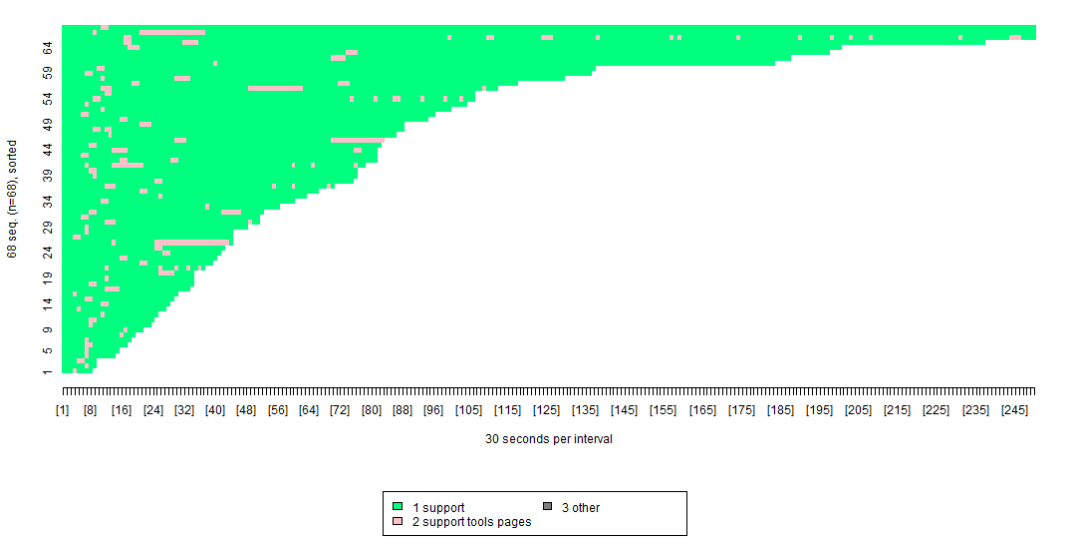
Visualization of reuse of the support tools in relation to the session on getting support.

We also used the visualization tool to provide a detailed breakdown of how the “support” tools were reused. Figure 6 shows that 68/104 participants (ie, 65% of those who were able to reaccess them) reused the tools in the “Getting Support” subcategory, which included information about the importance of getting support from others when trying to lose weight, and the various ways in which participants could get support from their nurse.

In Figure 6, each color represents a separate group of pages. For example, the light green stands for the support pages, and the pink stands for the support tools pages. The x-axis shows the length of time spent viewing each group of pages, broken down into blocks of 30 seconds. The y-axis can be thought of as a number of lines, each representing a specific participant. Participants are presented in order of how long they spent on the intervention, with those who spent less time nearer the bottom, and those who spent more time nearer the top.

This visualization shows that although some participants reused the “getting support” tools all in one go after accessing the session on “getting support,” it was more common to follow each brief usage of the “getting support” session with reuse of the “getting support” tools. Table 6 provides a precise breakdown of the proportion of participants using each of the support tools.

**Table 6:** Numbers of participants who reused the support tools.

Support topic	Numbers viewed n (%)
Getting support	68 (65.3)
Sending motivational emails	3 (2.8)
Ask the nurse	6 (5.8)
Social times	1 (0.9)

**Figure 7:**
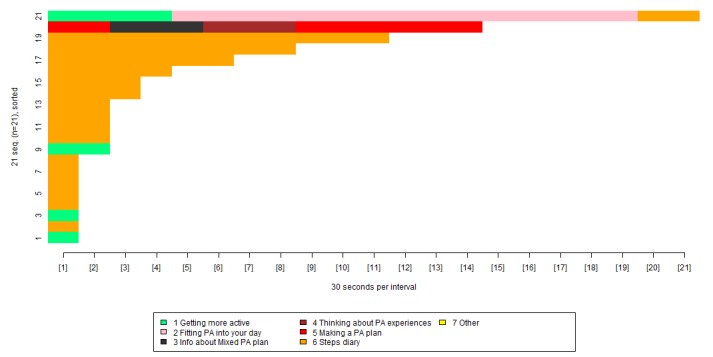
Visualization of participants’ repeated use of the physical activity plan tools.

Finally, we carried out a visualization to examine how participants reused the physical activity plan tools, as shown in Figure 7.

In Figure 7, each color represents a separate group of pages. For example, the orange shows usage of the steps diary and the light green shows usage of pages on getting more active. The x-axis shows the length of time spent viewing each group of pages, broken down into blocks of 30 seconds. The y-axis can be thought of as a number of lines, each representing a specific participant. Participants are presented in order of how long they spent on the intervention, with those who spent less time nearer the bottom, and those who spent more time nearer the top.

Figure 7 shows that the most widely reused physical activity tools pages were the steps diary (orange) and the pages on getting more active (light green), but that some of the other tools were used only by one person. Table 7 provides a precise breakdown of the proportion of participants using each of the physical activity tools.

**Table 7:** Numbers (and percentages) of participants who used the physical activity tools.

Physical activity topic	Numbers viewed n (%)
Getting more active	4 (3.0)
Thinking about fitting physical activity into your day	1 (0.8)
Information about the walking plan	0 (0)
Information about the mixed physical activity plan	1 (0.8)
Thinking about your walking experiences	0 (0)
Thinking about your physical activity experiences	1 (0.8)
Making a detailed walking plan	0 (0)
Making a detailed physical activity plan	1 (0.8)
Steps diary	17 (12.9)

### Patient Characteristics Related to Web Usage

Using the visualization tool, we were able to download the IDs of participants who followed particular usage patterns. This enabled the creation of a new usage variable that detailed who had/had not used particular intervention components and could be followed up with further statistical analysis using SPSS.

### Usage of Optional Sessions

Sixty-two participants used both the goal and weight reviews and the optional sessions, but 58 accessed the goal and weight reviews but not the optional sessions. Participants who did not use the optional sessions had a higher BMI at baseline (36.68 vs 34.60), were more likely to use the low-carbohydrate plan (χ^2^_2_=8.71, *P=*.03) and were more likely to use the walking plan (χ^2^_2_=2.08, *P*<.001). For these analyses, participants were classified as using the last plan they used. There was no difference in weight loss (kilograms) between those who used the optional sessions and those who did not, 3.67kg (SD 6.42) versus 2.14kg (SD 4.75; *t*_134_=1.54, *P*=.13).

### Repeated Use of Eating Plan Tools

Overall, 106 participants reused the eating plan tools, of whom 76 reused the weekly food diary. Participants who reused the weekly food diary were older, 53.62 versus 48.95 (*t*_134_=−2.11, *P*=.04), and completed more goal and weight review sessions than those who did not reuse the diary, 8.89 versus 3.23 (*t*_125.34_=−3.64, *P*<.001). There was no difference in weight loss between those who did and did not reuse the food diary (2.95, SD 5.53) versus (3.11, SD 6.17; *t*_134_=0.16, *P*=.87).

### Repeated Use of Physical Activity Tools

Overall, 21 participants reused the physical activity tools. Those who reused the steps diary were older than those who did not, 58.82 (SD 14.44) versus 50.52 (SD 12.45; *t*_134_=−2.52, *P*=.01). Participants who reused the steps diary (physical activity plan tools; n=17), lost more weight than those who did not, 5.78 kg (SD 6.87) versus 2.63 kg (SD 5.56; *t*_134_=−2.12, *P*=.04).

### Repeated Use of Getting Support Tools

Use of getting support tools was analyzed for the nurse groups only (as the Web group did not receive nurse support). A total of 68/104 participants (65% of those who were able to reaccess them) reused the getting support tools. There were no differences at baseline between those who did and did not use the getting support tools. However, those who used the getting support tools completed more of the sessions than those who did not, 3.39 (SD 1.14) versus 0.5 (SD 0.59; *t*_77.48_=−15.68, *P*<.001), and more goal and weight reviews than those who did not, 0 (SD 0) versus 8.81 (SD 10.65; *t*_66_=−6.77, *P*<.001). They also lost more weight than those who did not, 4.03 kg (SD 6.93) versus 1.53 kg (SD 4.04; *t*_70.04_=−2.12, *P*=.038).

## Discussion

### Principal Findings

This paper had three main aims, which the visualization tool was able to help us realize. These were as follows: (1) to see patterns of Web usage, (2) to carry out a moderator analysis of patient characteristics related to Web usage, and (3) to determine which pages were related to benefit from the Web-based intervention. These results are discussed in the following section in relation to these aims.

First, the visualization tool was extremely helpful in enabling us to determine patterns of Web usage. A first key observation is that the vast majority of participants who went online accessed the first session, but there was a drop of approximately 20% of participants from the first session (n=132 in part 1 and n=120 in part 2) to the second session (n=104). This is similar to the rapid attrition rate reported in similar Web-based weight loss interventions [3-5]. Dropout then continued at a rate of approximately 10% per session. Breaking down the first session into two parts based on content covered (as it was very long and each part had a similar length to the other full sessions) and checking how many participants accessed the last page of session 1 enabled us to see that almost 90% of participants completed the first session (n=120/132). To ensure all essential information is covered, each session should be presented as early as possible in the intervention. Interventions that aim to also prioritize physical activity should present this as early on as possible.

A second key observation is that only half the participants accessed any of the optional sessions, and each optional session was viewed by less than 25% of participants. Nevertheless, nearly half the participants continued to use the weekly goal and weight review, despite deciding not to access new optional content. In retrospect, this pattern of usage could have been unintentionally prompted by the design of the page following goal review, as the logout option was prominently placed. Alternatively, it could mean that participants felt the additional sessions were neither necessary nor particularly novel (as they covered topics that are commonly addressed by other weight management interventions). In support of this interpretation, there were no differences in weight loss between participants who did and did not use the optional sessions, indicating that the optional content was indeed not necessary for weight loss. In addition, those who chose not to access the optional content had a higher BMI at baseline, so may have been more likely to have encountered similar content in previous weight management attempts. This finding justifies the decision to make these sessions optional, and also suggests that for many participants the goal and weight review (which provided individualized progress-relevant feedback messages as well as a weight loss graph) was more important and rewarding to access than the generic weight management advice.

The eating plan tools were the most reused, especially the weekly food diary, and information about the low-calorie eating plan and the low-carbohydrate eating plan. Thus, explorations of usage patterns using visualization tools can help to identify the particular intervention tools that participants are keen to reuse online. Such insights can help design hybrid interventions that enable access to selected intervention content through multiple digital devices (eg, mobile phone apps). For example, a mixed-methods evaluation of a supplemental POWeR mobile phone app also showed that participants particularly valued being able to reaccess food lists associated with their eating plans on the go via their mobile phone [17]. The eating plan tools were the most basic weight management tools, and less essential tools such as the motivational “reasons to lose weight” or “sending motivational emails” support tools were not reused. However, this does not necessarily mean that the less essential tools were not valued by participants. It could be that participants engaged with these tools at their first presentation during the core session (eg, by printing out their reasons to lose weight card or setting up support emails there and then) and did not need to reuse them via the POWeR website.

Those who reused the food diary were older and had completed more goal and weight reviews than those who had not. It is possible that these participants may have been more conscientious in their attitude to weight loss, or that younger participants could have been using alternative tools. However, it is important to note that those who reused the food diary did not lose more weight than other participants. To minimize the intrusiveness and burden of weight management, POWeR specifically encourages users to employ food diaries only occasionally, as diagnostic tools when necessary, and not to rely on them for long-term weight management[18].

Those who reused the getting support tools had completed more sessions and goal and weight reviews and lost more weight than those who did not. This suggests that the support tools were helpful in enabling weight loss. The challenge now is engaging with those users who did not use the support tools. Interestingly, very few people reused the “ask the nurse” function, which allowed users to send queries or messages to the nurse providing them with support. Some POWeR users have indicated in our follow-up interviews that they would like to be able to access human support when they feel the need [19], but it appears that the facility to send the nurse an email may not meet this need. This could be because email is an insufficiently personal medium to access support [20], but it could also indicate that the opportunity to contact the nurse should be presented differently in future interventions; for example, perhaps offered as an immediate option in goal feedback if participants are not meeting their goals (rather than requiring users to access the option from their tools). Alternatively, these findings may indicate that people did not feel the need to contact the nurse, although they felt that it was helpful to have the option there.

Very few people reused the physical activity tools, suggesting that physical activity may not have been seen as an important part of weight management by POWeR users. However, of the physical activity tools, the steps diary was the most widely reused, and was associated with greater weight loss. Users of the steps diary may have used pedometers. They may also have had increased levels of autonomous motivation as this has mediated the effect of self-monitoring and diary usage on weight loss in previous studies [21]. It may therefore be beneficial to find ways to increase repeated and regular usage of the steps diary [22]. It is important to note that participants could only reuse the steps diary if they had chosen to follow the walking plan. From these results it is therefore not clear whether it was specifically the steps diary that was useful, or whether the walking plan was more beneficial than the mixed physical activity plan.

### Limitations

This study had several limitations. First, the results described here are based on a single feasibility study, and it is unclear how widely they would apply to a wider population. In particular, the sample participating in POWeR included fewer men and very few members of ethnic minorities. However, the sample was not young or highly educated, and as such could be considered broadly representative of the population eligible to enroll in such an intervention in primary care [11]. Second, although our exploratory analyses identified a number of possible patterns in Web usage and associations with outcome, further research is needed to confirm these patterns and test the hypotheses arising from this study. Third, the results regarding use of the steps diary and weight loss were based on a small number of users of the steps diary, and should therefore be interpreted with caution. This needs to be replicated with larger populations. Fourth, we considered the intervention groups from POWeR as a single population. It is possible that nurse support may have influenced Web usage. We were not able to determine this due to the small sample size.

### Conclusions

The visualization tool provided a useful and efficient method for interpreting and exploring a very large dataset on usage of a Web-based weight management intervention. Specifically, the visualization tool helped to determine aspects of the intervention design and content that seem to encourage and discourage repeated use. Insights gained from a visual analysis of usage data also helped to determine the associations between usage patterns, participant characteristics, and weight change in subsequent statistical analyses. The visualization tool complements the work of Morrison and Doherty [15] by enabling an in-depth analysis of all participants’ usage of EVERY intervention component within one sequence plot. Different visualization tools are likely to be more or less useful depending on the intervention architecture and research questions of interest. The visualization tool presented here may be particularly useful for inductive analyses of tunneled interventions. By contrast, the toolkits developed by van Gemert-Pijnen and colleagues [12] may be particularly beneficial for usage analyses following a priori assumptions about key intervention components. The toolkit developed by Morrison and Doherty[15] may be particularly beneficial for individual-level analyses or group-level analyses of nontunneled interventions that do not have a clear start and end point. Visualization toolkits can be used as part of a mixed-methods approach for developing and evaluating digital interventions that seek to arrive at a more complete picture of the differences in the way in which participants use an intervention, supplemented by qualitative insights about participants’ subjective experiences of using the intervention [23] and quantitative data on the effect of the intervention on health-related outcomes. Further usage of visualization techniques is highly recommended in order to (1) guide the design (redesign) of future interventions so that they enable easy access to valued intervention content, and (2) unlock the active ingredients in Web-based interventions, so they can be enhanced to reach and engage the maximum eligible population.

## Abbreviations

BMI: body mass index
POWeR: Positive Online Weight Reduction
